# 1-Nitro-4-(2-nitro­prop-1-en­yl)benzene

**DOI:** 10.1107/S1600536810023676

**Published:** 2010-06-26

**Authors:** Jian-Ke Yang, Mei Zheng, Shu-Ping Luo, Zhao-Bo Li

**Affiliations:** a Hangzhou Zhongmei Huadong Pharmaceutical Co. Ltd, Hangzhou, 310000, People’s Republic of China; b State Key Laboratory Breeding Base of Green Chemistry-Synthesis Technology, Zhejiang University of Technology, Hangzhou, 310014, People’s Republic of China

## Abstract

The asymmetric unit of the title compound, C_9_H_8_N_2_O_4_, contains two crystallographically independent mol­ecules, both of which adopt an *E* configuration about the C=C bond. In the crystal, the mol­ecules stack into columns along the *c* axis through π–π inter­actions, with centroid–centroid distances of 3.695 (3) and 3.804 (3) Å. The columns are further connected into a three-dimensional network by C—H⋯O hydrogen bonds.

## Related literature

For background to the chemistry of nitro­alkenes, see: Ballini & Petrini (2004 ▶); Berner *et al.* (2002 ▶); Ono (2001 ▶).
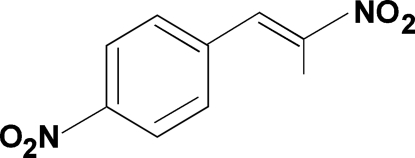


## Experimental

### 

#### Crystal data

C_9_H_8_N_2_O_4_
*M* *_r_* = 208.17
Monoclinic, 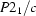

*a* = 13.3621 (11) Å
*b* = 9.7648 (7) Å
*c* = 14.8835 (11) Å
β = 91.290 (2)°
*V* = 1941.5 (3) Å^3^
*Z* = 8
Mo *K*α radiation
μ = 0.11 mm^−1^
*T* = 296 K
0.38 × 0.29 × 0.20 mm

#### Data collection

Rigaku R-AXIS RAPID diffractometer
Absorption correction: multi-scan (*ABSCOR*; Higashi, 1995 ▶) *T*_min_ = 0.947, *T*_max_ = 0.978
18620 measured reflections
4430 independent reflections
1883 reflections with *I* > 2σ(*I*)
*R*_int_ = 0.042

#### Refinement

*R*[*F* ^2^ > 2σ(*F* ^2^)] = 0.053
*wR*(*F* ^2^) = 0.168
*S* = 1.01
4430 reflections
273 parameters
H-atom parameters constrained
Δρ_max_ = 0.24 e Å^−3^
Δρ_min_ = −0.17 e Å^−3^

### 

Data collection: *PROCESS-AUTO* (Rigaku, 2006 ▶); cell refinement: *PROCESS-AUTO*; data reduction: *CrystalStructure* (Rigaku, 2007 ▶); program(s) used to solve structure: *SHELXS97* (Sheldrick, 2008 ▶); program(s) used to refine structure: *SHELXL97* (Sheldrick, 2008 ▶); molecular graphics: *ORTEP-3 for Windows* (Farrugia, 1997 ▶); software used to prepare material for publication: *WinGX* (Farrugia, 1999 ▶).

## Supplementary Material

Crystal structure: contains datablocks I. DOI: 10.1107/S1600536810023676/rz2463sup1.cif

Structure factors: contains datablocks I. DOI: 10.1107/S1600536810023676/rz2463Isup2.hkl

Additional supplementary materials:  crystallographic information; 3D view; checkCIF report

## Figures and Tables

**Table 1:** Hydrogen-bond geometry (Å, °)

*D*—H⋯*A*	*D*—H	H⋯*A*	*D*⋯*A*	*D*—H⋯*A*
C9*B*—H18⋯O4*A*^i^	0.93	2.55	3.386 (4)	149

Symmetry code: (i) 
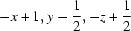
.

**Fig. 1.**
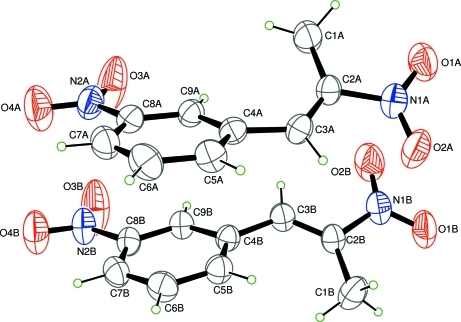
The asymmetric unit of the title compound with the atomic labeling scheme; displacement ellipsoids are drawn at the 50% probability level.

**Fig. 2.**
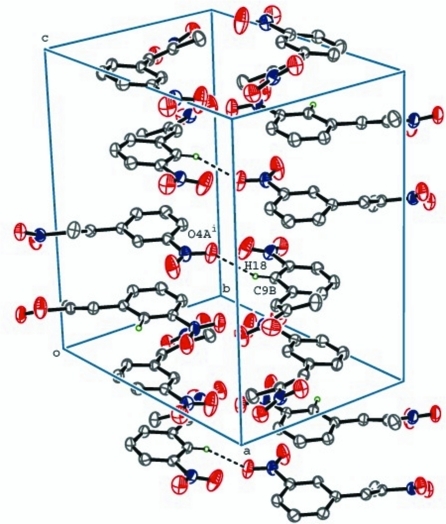
Crystal packing of the title compound showing the intermolecular hydrogen interaction dash lines. Displacement ellipsoids are drawn at the 50% probability level.

## supplementary crystallographic information

### Comment

Nitroalkenes are important organic intermediates, since they can be converted to synthetically useful N– and O-containing organic molecules, such as amines, aldehydes, carboxylic acids, or denitrated compounds (Ono, 2001; Berner *et al.*, 2002; Ballini & Petrini, 2004). As a contribution in this field, we have synthesized a series of nitroalkenes by employing benzaldehydes and nitroethane. We report here the crystal structure of the title compound.

The asymmetric unit of the title compound (Fig. 1) contains two crystallographically independent molecules. Both molecules adopt an *E* configuration with respect to the C═C double bond. In the crystal packing (Fig. 2), the molecules interact through π···π interactions (centroid-to-centroid distances of 3.695 (3) and 3.804 (3) Å) to form columns along the *c* axis. The column are further connected into a three-dimensional network by C—H···O hydrogen bonds (Table 1).

### Experimental

To a solution of 3-nitro-benzaldehyde (50 mmol) in AcOH (25 mL), nitroethane (75 mmol) was added, followed by butylamine (100 mmol, 7.4 mL). The mixture was sonicated at 60 °C, until GC showed full conversion of the aldehyde. The mixture was poured into ice water, the precipitate was filtered off, washed with water and recrystallized from EtOH/EtOAc to give the product. Single crystals were obtained by slow evaporation of an cyclohexane-EtOAc (10:1 *v*/*v*) solution.

### Refinement

All H atoms were placed in calculated positions and refined using a riding model, with C—H = 0.93-0.96 Å, and with *U*_iso_(H) = 1.2 *U*_eq_(C) or 1.5 *U*_eq_(C) for methyl H atoms.

### Crystal data

**Table:**

C_9_H_8_N_2_O_4_	*F*(000) = 864
*M**_r_* = 208.17	*D*_x_ = 1.424 Mg m^−^^3^
Monoclinic, *P*2_1_/*c*	Mo *K*α radiation, λ = 0.71073 Å
Hall symbol: -P 2ybc	Cell parameters from 9423 reflections
*a* = 13.3621 (11) Å	θ = 3.0–27.5°
*b* = 9.7648 (7) Å	µ = 0.11 mm^−^^1^
*c* = 14.8835 (11) Å	*T* = 296 K
β = 91.290 (2)°	Chunk, yellow
*V* = 1941.5 (3) Å^3^	0.38 × 0.29 × 0.20 mm
*Z* = 8	

### Data collection

**Table:**

Rigaku R-AXIS RAPID diffractometer	4430 independent reflections
Radiation source: rolling anode	1883 reflections with *I* > 2σ(*I*)
graphite	*R*_int_ = 0.042
Detector resolution: 10.00 pixels mm^-1^	θ_max_ = 27.5°, θ_min_ = 3.1°
ω scans	*h* = −17→17
Absorption correction: multi-scan (*ABSCOR*; Higashi, 1995)	*k* = −12→11
*T*_min_ = 0.947, *T*_max_ = 0.978	*l* = −19→19
18620 measured reflections	

### Refinement

**Table:**

Refinement on *F*^2^	Primary atom site location: structure-invariant direct methods
Least-squares matrix: full	Secondary atom site location: difference Fourier map
*R*[*F*^2^ > 2σ(*F*^2^)] = 0.053	Hydrogen site location: inferred from neighbouring sites
*wR*(*F*^2^) = 0.168	H-atom parameters constrained
*S* = 1.01	*w* = 1/[σ^2^(*F*_o_^2^) + (0.0633*P*)^2^ + 0.4203*P*] where *P* = (*F*_o_^2^ + 2*F*_c_^2^)/3
4430 reflections	(Δ/σ)_max_ = 0.001
273 parameters	Δρ_max_ = 0.24 e Å^−^^3^
0 restraints	Δρ_min_ = −0.17 e Å^−^^3^

### Special details

**Table:**

Geometry. All e.s.d.'s (except the e.s.d. in the dihedral angle between two l.s. planes) are estimated using the full covariance matrix. The cell e.s.d.'s are taken into account individually in the estimation of e.s.d.'s in distances, angles and torsion angles; correlations between e.s.d.'s in cell parameters are only used when they are defined by crystal symmetry. An approximate (isotropic) treatment of cell e.s.d.'s is used for estimating e.s.d.'s involving l.s. planes.
Refinement. Refinement of *F*^2^ against ALL reflections. The weighted *R*-factor *wR* and goodness of fit *S* are based on *F*^2^, conventional *R*-factors *R* are based on *F*, with *F* set to zero for negative *F*^2^. The threshold expression of *F*^2^ > σ(*F*^2^) is used only for calculating *R*-factors(gt) *etc*. and is not relevant to the choice of reflections for refinement. *R*-factors based on *F*^2^ are statistically about twice as large as those based on *F*, and *R*- factors based on ALL data will be even larger.

### Fractional atomic coordinates and isotropic or equivalent isotropic displacement parameters (Å^2^)

**Table:**

	*x*	*y*	*z*	*U*_iso_*/*U*_eq_	
N1A	0.91640 (18)	0.3305 (3)	0.05652 (17)	0.0806 (6)	
O2A	0.98875 (16)	0.3688 (2)	0.10125 (17)	0.1116 (8)	
O1A	0.90989 (18)	0.2158 (2)	0.02430 (17)	0.1120 (8)	
C4A	0.77860 (18)	0.6710 (2)	0.05586 (16)	0.0625 (6)	
O1B	0.96098 (19)	0.2340 (2)	0.31850 (17)	0.1199 (8)	
N1B	0.8836 (2)	0.2846 (3)	0.29171 (18)	0.0892 (7)	
C3A	0.84789 (17)	0.5546 (3)	0.06249 (16)	0.0678 (7)	
H3	0.9118	0.5744	0.0849	0.081*	
C8B	0.59839 (19)	0.7663 (3)	0.31450 (16)	0.0679 (7)	
C2A	0.83112 (17)	0.4254 (3)	0.04069 (16)	0.0658 (6)	
C2B	0.86722 (19)	0.4327 (2)	0.30718 (17)	0.0673 (6)	
C3B	0.77664 (18)	0.4791 (2)	0.28873 (17)	0.0692 (7)	
H12	0.7304	0.4142	0.2686	0.083*	
C9A	0.67661 (18)	0.6603 (2)	0.06964 (16)	0.0650 (6)	
H9	0.6480	0.5753	0.0809	0.078*	
O2B	0.8201 (2)	0.2192 (2)	0.2511 (2)	0.1380 (11)	
C4B	0.73898 (18)	0.6202 (2)	0.29576 (16)	0.0617 (6)	
C5B	0.7965 (2)	0.7370 (3)	0.28421 (18)	0.0723 (7)	
H14	0.8646	0.7281	0.2739	0.087*	
C9B	0.63788 (18)	0.6371 (2)	0.31073 (16)	0.0654 (6)	
H18	0.5970	0.5611	0.3182	0.078*	
C5A	0.8177 (2)	0.8007 (3)	0.04022 (18)	0.0761 (7)	
H5	0.8861	0.8103	0.0316	0.091*	
C6B	0.7547 (2)	0.8658 (3)	0.28776 (19)	0.0815 (8)	
H15	0.7948	0.9426	0.2798	0.098*	
N2B	0.4902 (2)	0.7794 (3)	0.32987 (19)	0.0929 (8)	
N2A	0.51053 (19)	0.7625 (3)	0.08429 (18)	0.0910 (7)	
C8A	0.6181 (2)	0.7759 (3)	0.06654 (17)	0.0702 (7)	
O3A	0.47832 (17)	0.6518 (3)	0.0982 (3)	0.1743 (15)	
O4A	0.45844 (19)	0.8608 (3)	0.0844 (2)	0.1402 (10)	
O3B	0.4434 (2)	0.6811 (3)	0.3488 (3)	0.1726 (14)	
C7B	0.6543 (2)	0.8819 (3)	0.30301 (18)	0.0791 (8)	
H16	0.6254	0.9685	0.3054	0.095*	
C1B	0.9567 (2)	0.5036 (3)	0.3453 (2)	0.0955 (9)	
H10A	1.0044	0.5176	0.2989	0.143*	
H10B	0.9864	0.4486	0.3923	0.143*	
H10C	0.9373	0.5906	0.3694	0.143*	
C7A	0.6564 (2)	0.9036 (3)	0.05062 (18)	0.0830 (8)	
H7	0.6152	0.9804	0.0489	0.100*	
C6A	0.7576 (2)	0.9148 (3)	0.0372 (2)	0.0894 (9)	
H6	0.7855	1.0002	0.0261	0.107*	
C1A	0.7418 (2)	0.3607 (3)	−0.0012 (2)	0.0956 (10)	
H1A	0.6986	0.4302	−0.0261	0.143*	
H1B	0.7067	0.3097	0.0433	0.143*	
H1C	0.7621	0.3000	−0.0482	0.143*	
O4B	0.45068 (18)	0.8883 (3)	0.3199 (2)	0.1367 (10)	

### Atomic displacement parameters (Å^2^)

**Table:**

	*U*^11^	*U*^22^	*U*^33^	*U*^12^	*U*^13^	*U*^23^
N1A	0.0647 (15)	0.0786 (16)	0.0986 (17)	0.0075 (12)	0.0091 (13)	0.0115 (14)
O2A	0.0697 (14)	0.1051 (16)	0.159 (2)	0.0148 (12)	−0.0217 (14)	0.0049 (14)
O1A	0.1112 (18)	0.0803 (14)	0.144 (2)	0.0245 (12)	0.0023 (15)	−0.0107 (14)
C4A	0.0590 (15)	0.0640 (14)	0.0643 (15)	−0.0041 (12)	−0.0004 (11)	−0.0001 (12)
O1B	0.1121 (19)	0.1086 (17)	0.139 (2)	0.0557 (14)	−0.0007 (15)	−0.0028 (14)
N1B	0.0780 (18)	0.0757 (16)	0.115 (2)	0.0129 (14)	0.0161 (15)	−0.0010 (14)
C3A	0.0511 (14)	0.0744 (17)	0.0777 (16)	−0.0019 (12)	0.0001 (12)	0.0052 (13)
C8B	0.0631 (16)	0.0704 (16)	0.0707 (16)	0.0113 (13)	0.0084 (12)	0.0049 (13)
C2A	0.0531 (14)	0.0678 (15)	0.0768 (16)	0.0032 (12)	0.0079 (12)	0.0024 (13)
C2B	0.0622 (16)	0.0583 (14)	0.0819 (17)	0.0033 (12)	0.0101 (13)	0.0052 (12)
C3B	0.0590 (16)	0.0599 (14)	0.0889 (18)	−0.0035 (12)	0.0080 (13)	0.0000 (13)
C9A	0.0589 (16)	0.0623 (14)	0.0737 (16)	−0.0003 (12)	−0.0039 (12)	0.0008 (12)
O2B	0.1084 (19)	0.0716 (14)	0.233 (3)	0.0038 (13)	−0.014 (2)	−0.0303 (16)
C4B	0.0582 (15)	0.0591 (14)	0.0679 (15)	0.0006 (11)	0.0022 (11)	0.0044 (11)
C5B	0.0627 (16)	0.0686 (17)	0.0855 (18)	−0.0032 (13)	0.0017 (13)	0.0030 (13)
C9B	0.0623 (16)	0.0596 (14)	0.0746 (16)	0.0002 (12)	0.0100 (12)	0.0079 (12)
C5A	0.0719 (17)	0.0695 (17)	0.0874 (18)	−0.0088 (14)	0.0090 (14)	0.0009 (14)
C6B	0.085 (2)	0.0610 (16)	0.099 (2)	−0.0130 (14)	0.0015 (16)	0.0029 (14)
N2B	0.0783 (18)	0.0790 (17)	0.122 (2)	0.0212 (15)	0.0218 (15)	0.0153 (15)
N2A	0.0680 (17)	0.0898 (18)	0.115 (2)	0.0143 (15)	−0.0088 (14)	0.0025 (15)
C8A	0.0630 (17)	0.0761 (17)	0.0713 (16)	0.0042 (13)	−0.0036 (13)	−0.0032 (13)
O3A	0.0603 (15)	0.124 (2)	0.339 (5)	0.0037 (15)	0.001 (2)	0.063 (3)
O4A	0.0951 (17)	0.1155 (19)	0.211 (3)	0.0362 (15)	0.0182 (17)	−0.0182 (18)
O3B	0.0914 (19)	0.124 (2)	0.306 (4)	0.0254 (16)	0.081 (2)	0.067 (2)
C7B	0.091 (2)	0.0597 (15)	0.0869 (19)	0.0108 (15)	0.0042 (16)	0.0013 (13)
C1B	0.0658 (18)	0.090 (2)	0.130 (3)	−0.0040 (15)	−0.0178 (17)	0.0151 (18)
C7A	0.095 (2)	0.0683 (18)	0.0855 (19)	0.0133 (16)	0.0029 (16)	0.0008 (14)
C6A	0.104 (2)	0.0625 (17)	0.103 (2)	−0.0088 (16)	0.0148 (18)	0.0029 (15)
C1A	0.0722 (19)	0.085 (2)	0.130 (3)	0.0024 (15)	−0.0080 (17)	−0.0250 (18)
O4B	0.0992 (18)	0.0986 (17)	0.213 (3)	0.0340 (14)	0.0132 (17)	0.0055 (17)

### Geometric parameters (Å, °)

**Table:**

N1A—O2A	1.220 (3)	C5B—C6B	1.378 (4)
N1A—O1A	1.220 (3)	C5B—H14	0.9300
N1A—C2A	1.484 (3)	C9B—H18	0.9300
C4A—C9A	1.387 (3)	C5A—C6A	1.374 (4)
C4A—C5A	1.392 (3)	C5A—H5	0.9300
C4A—C3A	1.468 (3)	C6B—C7B	1.375 (4)
O1B—N1B	1.206 (3)	C6B—H15	0.9300
N1B—O2B	1.212 (3)	N2B—O3B	1.183 (3)
N1B—C2B	1.482 (3)	N2B—O4B	1.195 (3)
C3A—C2A	1.320 (3)	N2A—O3A	1.184 (3)
C3A—H3	0.9300	N2A—O4A	1.186 (3)
C8B—C7B	1.366 (4)	N2A—C8A	1.474 (4)
C8B—C9B	1.369 (3)	C8A—C7A	1.370 (4)
C8B—N2B	1.474 (4)	C7B—H16	0.9300
C2A—C1A	1.476 (3)	C1B—H10A	0.9600
C2B—C3B	1.315 (3)	C1B—H10B	0.9600
C2B—C1B	1.484 (3)	C1B—H10C	0.9600
C3B—C4B	1.471 (3)	C7A—C6A	1.376 (4)
C3B—H12	0.9300	C7A—H7	0.9300
C9A—C8A	1.373 (3)	C6A—H6	0.9300
C9A—H9	0.9300	C1A—H1A	0.9600
C4B—C9B	1.384 (3)	C1A—H1B	0.9600
C4B—C5B	1.388 (3)	C1A—H1C	0.9600
			
O2A—N1A—O1A	123.0 (2)	C6A—C5A—C4A	121.4 (3)
O2A—N1A—C2A	119.4 (2)	C6A—C5A—H5	119.3
O1A—N1A—C2A	117.6 (2)	C4A—C5A—H5	119.3
C9A—C4A—C5A	117.9 (2)	C7B—C6B—C5B	120.6 (3)
C9A—C4A—C3A	123.5 (2)	C7B—C6B—H15	119.7
C5A—C4A—C3A	118.5 (2)	C5B—C6B—H15	119.7
O1B—N1B—O2B	122.2 (3)	O3B—N2B—O4B	121.1 (3)
O1B—N1B—C2B	118.5 (3)	O3B—N2B—C8B	119.4 (3)
O2B—N1B—C2B	119.2 (3)	O4B—N2B—C8B	119.4 (3)
C2A—C3A—C4A	128.3 (2)	O3A—N2A—O4A	121.6 (3)
C2A—C3A—H3	115.8	O3A—N2A—C8A	118.2 (3)
C4A—C3A—H3	115.8	O4A—N2A—C8A	120.2 (3)
C7B—C8B—C9B	123.0 (2)	C7A—C8A—C9A	122.7 (3)
C7B—C8B—N2B	119.2 (2)	C7A—C8A—N2A	118.7 (3)
C9B—C8B—N2B	117.8 (2)	C9A—C8A—N2A	118.5 (3)
C3A—C2A—C1A	130.1 (2)	C8B—C7B—C6B	117.7 (2)
C3A—C2A—N1A	115.6 (2)	C8B—C7B—H16	121.2
C1A—C2A—N1A	114.2 (2)	C6B—C7B—H16	121.2
C3B—C2B—N1B	116.2 (2)	C2B—C1B—H10A	109.5
C3B—C2B—C1B	130.5 (2)	C2B—C1B—H10B	109.5
N1B—C2B—C1B	113.2 (2)	H10A—C1B—H10B	109.5
C2B—C3B—C4B	128.5 (2)	C2B—C1B—H10C	109.5
C2B—C3B—H12	115.8	H10A—C1B—H10C	109.5
C4B—C3B—H12	115.8	H10B—C1B—H10C	109.5
C8A—C9A—C4A	119.5 (2)	C8A—C7A—C6A	118.0 (3)
C8A—C9A—H9	120.2	C8A—C7A—H7	121.0
C4A—C9A—H9	120.2	C6A—C7A—H7	121.0
C9B—C4B—C5B	117.8 (2)	C7A—C6A—C5A	120.5 (3)
C9B—C4B—C3B	117.4 (2)	C7A—C6A—H6	119.8
C5B—C4B—C3B	124.7 (2)	C5A—C6A—H6	119.8
C6B—C5B—C4B	121.3 (3)	C2A—C1A—H1A	109.5
C6B—C5B—H14	119.4	C2A—C1A—H1B	109.5
C4B—C5B—H14	119.4	H1A—C1A—H1B	109.5
C8B—C9B—C4B	119.7 (2)	C2A—C1A—H1C	109.5
C8B—C9B—H18	120.2	H1A—C1A—H1C	109.5
C4B—C9B—H18	120.2	H1B—C1A—H1C	109.5
			
C9A—C4A—C3A—C2A	34.4 (4)	C5B—C4B—C9B—C8B	−0.6 (4)
C5A—C4A—C3A—C2A	−149.6 (3)	C3B—C4B—C9B—C8B	−177.6 (2)
C4A—C3A—C2A—C1A	4.1 (5)	C9A—C4A—C5A—C6A	−0.8 (4)
C4A—C3A—C2A—N1A	−179.1 (2)	C3A—C4A—C5A—C6A	−177.1 (2)
O2A—N1A—C2A—C3A	12.3 (4)	C4B—C5B—C6B—C7B	0.0 (4)
O1A—N1A—C2A—C3A	−168.5 (3)	C7B—C8B—N2B—O3B	−172.5 (3)
O2A—N1A—C2A—C1A	−170.5 (3)	C9B—C8B—N2B—O3B	8.8 (4)
O1A—N1A—C2A—C1A	8.8 (4)	C7B—C8B—N2B—O4B	10.2 (4)
O1B—N1B—C2B—C3B	−170.4 (3)	C9B—C8B—N2B—O4B	−168.6 (3)
O2B—N1B—C2B—C3B	11.1 (4)	C4A—C9A—C8A—C7A	−0.3 (4)
O1B—N1B—C2B—C1B	7.1 (4)	C4A—C9A—C8A—N2A	−178.0 (2)
O2B—N1B—C2B—C1B	−171.3 (3)	O3A—N2A—C8A—C7A	−179.8 (3)
N1B—C2B—C3B—C4B	−178.8 (2)	O4A—N2A—C8A—C7A	0.3 (4)
C1B—C2B—C3B—C4B	4.2 (5)	O3A—N2A—C8A—C9A	−2.0 (4)
C5A—C4A—C9A—C8A	0.7 (3)	O4A—N2A—C8A—C9A	178.1 (3)
C3A—C4A—C9A—C8A	176.7 (2)	C9B—C8B—C7B—C6B	−0.5 (4)
C2B—C3B—C4B—C9B	−152.8 (3)	N2B—C8B—C7B—C6B	−179.3 (2)
C2B—C3B—C4B—C5B	30.4 (4)	C5B—C6B—C7B—C8B	0.1 (4)
C9B—C4B—C5B—C6B	0.2 (4)	C9A—C8A—C7A—C6A	0.1 (4)
C3B—C4B—C5B—C6B	177.0 (2)	N2A—C8A—C7A—C6A	177.8 (2)
C7B—C8B—C9B—C4B	0.8 (4)	C8A—C7A—C6A—C5A	−0.2 (4)
N2B—C8B—C9B—C4B	179.5 (2)	C4A—C5A—C6A—C7A	0.6 (4)

### Hydrogen-bond geometry (Å, °)

**Table:**

*D*—H···*A*	*D*—H	H···*A*	*D*···*A*	*D*—H···*A*
C9B—H18···O4A^i^	0.93	2.55	3.386 (4)	149

Symmetry codes: (i) −*x*+1, *y*−1/2, −*z*+1/2.
